# Monoclonal B-cell lymphocytosis: a brief review for general clinicians

**DOI:** 10.1590/S1516-31802011000300008

**Published:** 2011-05-05

**Authors:** Daniel Mazza Matos, Roberto Passetto Falcão

**Affiliations:** I MD, PhD. Hematologist, Department of Clinical Medicine, Faculdade de Medicina de Ribeirão Preto, Universidade de São Paulo (FMRP/USP), Ribeirão Preto, São Paulo, Brazil.

**Keywords:** B-Lymphocytes, Leukemia, lymphocytic, chronic, B-cell, Flow cytometry, Epidemiology, Disease management, Linfócitos B, Leucemia linfocítica crônica de células B, Citometria de fluxo, Epidemiologia, Gerenciamento clínico

## Abstract

Monoclonal B-cell lymphocytosis (MBL) is a recently described medical condition that displays biological similarities to the most common subtype of adult leukemia in the Western world, i.e. chronic lymphocytic leukemia (CLL). Diagnostic criteria have been published with the aim of differentiating between these two entities. The overall prevalence of MBL is at least 100 times higher than that of CLL, which indirectly suggests that MBL is not necessarily a pre-leukemic condition, although in some circumstances, CLL cases can really be preceded by MBL. In view of this high prevalence rate, general clinicians and even non-hematological specialists have a high chance of being faced with individuals with MBL in their routine clinical practice. MBL is classified as “clinical MBL”, “population-screening MBL” and “atypical MBL” and the clinical management of affected individuals depends greatly on this differentiation. The present review provides a guide to diagnosing and following up MBL patients.

## RESUMO

A linfocitose monoclonal de células B (LMB) é uma condição médica recentemente descrita que exibe similaridades biológicas com o mais comum subtipo de leucemia em adultos de países ocidentais, qual seja, a leucemia linfocítica crônica (LLC). Critérios diagnósticos foram publicados com o intuito de separar as duas entidades. A prevalência global da LMB é pelo menos 100 vezes maior do que a da LLC, o que, indiretamente, sugere que a LMB não é necessariamente uma condição pré-leucêmica, embora, em algumas circunstâncias, casos de LLC possam realmente ser precedidos pela LMB. Em virtude dessa alta taxa de prevalência, clínicos gerais e mesmo outros especialistas não hematologistas têm grande chance de deparar-se com casos de LMB em suas rotinas clínicas. A LMB é classificada como “LMB clínica”, “LMB de *screening* populacional” e “LMB atípica”, sendo que o manuseio clínico dos indivíduos afetados depende substancialmente dessa diferenciação. A presente revisão fornece um guia para o diagnóstico e acompanhamento dos pacientes com LMB.

## DEFINITION AND HISTORY

Over the last few years, the more widespread availability of automated blood cell counters, associated with increasing technological advances in flow cytometry, has made it possible to recognize very low levels of circulating monoclonal B-lymphocytes that are immunophenotypically similar to chronic lymphocytic leukemia B-cells, in the peripheral blood of healthy subjects. Within this scenario, general clinicians and even non-hematological specialists have a high chance of coming across asymptomatic individuals with slight increases in absolute lymphocyte counts, composed of abnormal B-cell clones, in peripheral blood, but without the diagnostic criteria for chronic lymphocytic leukemia (CLL).^1^

The first published paper to describe these abnormal B-cells dates back to 1991, consisting of a report from a health study conducted by the Centers for Disease Control (CDC) in the United States, which detected monoclonal B-cells in people living near hazardous waste sites. In 1995, a public health service workshop in the United States put forward recommendations for case definitions and medical follow-up.^2^ Subsequently, over the next ten years, an increasing number of studies on different populations around the world, using different flow cytometry approaches, recognized that CD5+ and CD5− monoclonal B-lymphocytes could be found in individuals with no clinically apparent hematological disease.^3^

In 2005, the International Familial CLL Consortium proposed the term “monoclonal B-cell lymphocytosis” (MBL) to describe very low levels of circulating monoclonal B-cells that were identified by means of immunophenotypic characterization, in the peripheral blood of apparently health subjects. Furthermore, diagnostic criteria for this condition were defined and, with little modification, these continue to be used today (Chart 1).^4^

**Chart 1:** Diagnostic criteria and subclassification for monoclonal B-cell lymphocytosis (adapted from Shanafelt et al.)^15^

**DIAGNOSTIC CRITERIA**
Detection of a monoclonal B-cell population in the peripheral blood* **by at least one of following:** Light-chain restriction: overall kappa:lambda ratio > 3:1 or < 0.3:1, or > 25% of B cells lacking or expressing low levels of surface immunoglobulin† Heavy-chain monoclonal immunoglobulin heavy chain variable region (*IGHV*) rearrangements **Presence of a disease-specific immunophenotype**†,‡ **Absolute B-cell count < 5 x 10**^9^ **cells/l**† **No other features of a lymphoproliferative disorder:** Normal physical examination (no lymphadenopathy or organomegaly) Absence of B-symptoms (fever, weight loss or nighttime sweating) attributable to a non-Hodgkin lymphoma **No autoimmune or infectious disease**
**SUBCLASSIFICATION**
**CLL-like phenotype:** (A.1) Coexpression of CD5 with CD19, CD20^low^ and CD23† (A.2) Light-chain restriction with low surface immunoglobulin expression (very small MBL clones may be oligoclonal and thus not light-chain restricted)† **Atypical CLL phenotype:** (B.1) Coexpression of CD5 with CD19, but CD20^strong^ or CD23 negative† (B.2) Light-chain restriction with moderate/strong surface immunoglobulin expression† (B.3) Exclude t(11;14) to rule out mantle cell lymphoma **Non-CLL phenotype :** (C.1) CD5 negative† (C.2) Expression of CD20† (C.3) Light-chain restriction with moderate/strong surface immunoglobulin expression†

* When possible, a repeat assessment should demonstrate that MBL is stable over a three-month period;

† Defined by flow cytometry;

‡ A disease-specific immunophenotype is an abnormal B-cell phenotype that separates it from normal B-cells. The most common disease-specific immunophenotype is the presence of CD5+ B-lymphocytes with abnormally low expression of CD20 antigen.

## EPIDEMIOLOGY

The overall prevalence of MBL is at least 100 times higher than that of CLL, which indirectly suggests that MBL is not necessarily a pre-leukemic condition, but may represent an aspect of immunosenescence or the outcome from persistent immune stimulation (discussed later in “Biology and natural history of MBL”).^5^ Moreover, the prevalence of MBL is age-related and population-related. Rawstron et al.^6^ showed that the overall prevalence of CLL-like MBL among United Kingdom hospital outpatients without hematological or oncological diseases and with normal blood counts was 3.5%. The prevalence increased with age, from 2.1% in individuals between 40 and 60 years of age to 5.0% for individuals over 60 years of age. Ghia et al.^7^ studied individuals from a rural community referred for routine blood tests and showed that the overall prevalence was 5.1%. In a recent study conducted in a community in northern Italy, Dagklis et al.^8^ reported that the prevalence of CLL-like MBL and atypical MBL was 6.3%. The prevalence in individuals over 60 years of age was 8.9%. Other published papers have found different prevalence rates going from as low as 0.14%^9^ to as high as 12.0%,^10^ but this wide variation is probably related to the use of different flow cytometry approaches, i.e. other than the most commonly used four-color flow cytometry method. This hampers comparisons between these studies at the present time.

With regard to population subgroups, the highest prevalence of CLL-like MBL has been found in healthy first-degree relatives of patients with familial CLL, which is a condition characterized by the presence of two or more individuals with the diagnosis of CLL inside the same family.^11^ Thus, Rawstron et al.^12^ detected the presence of MBL in eight out of 59 individuals (13.5%) pertaining to 21 families with CLL in the United Kingdom. The prevalence in another study on 9 families with CLL in the United States was 18%.^13^ These studies clearly show that MBL is more frequently observed in CLL kindreds than in individuals from the general population.

Lastly, using the worldwide four-color flow cytometry approach, we have recently studied 167 healthy first-degree relatives of patients with just one case of CLL inside the family (sporadic CLL). We found seven CLL-like MBL cases (overall prevalence of 4.1%). However, the prevalence of individuals over 60 years of age was 15.6%, which suggests that in older first-degree relatives of patients with sporadic CLL, the risk of MBL detection is as high as in older first-degree relatives from CLL families.^14^ Nonetheless, at this moment, there is no evidence to suggest that searches for MBL should be conducted in first-degree relatives of patients with the diagnosis of CLL. However, it is recommended that relatives who are potential donors for allogeneic stem cell transplantation should be screened for MBL, mainly because the transplanted monoclonal B-cells could interfere with the residual disease monitoring performed by flow cytometry and, moreover, there is a possibility of expansion of the MBL clone in the recipient.^4^ Nevertheless, there is currently no consensus on whether or not matched relatives should be excluded, especially when there are no alternative donors inside the family.^15^

## BIOLOGY AND NATURAL HISTORY OF MBL

With regard to the phenotype determined by flow cytometry, monoclonal B-cell lymphocytosis has been classified as MBL with CLL-like phenotype, MBL with atypical CLL phenotype and MBL with non-CLL phenotype (Chart 1). Moreover, MBL can also be classified as cases detected in clinical practice (clinical MBL), in which individuals have absolute lymphocytosis, but with B-cell counts lower than 5 x 10^9^ cells/l, or otherwise, MBL cases accidentally found by screening individuals with a completely normal blood count (population-screening MBL).^15^

MBL with CLL-like phenotype accounts for most cases^8^ and exhibits a population of monoclonal B-cells that, in addition to CD5 and CD23 positivity, show low expression of CD20 and CD79b antigens. This profile is phenotypically identical to CLL B-cells.^16^ Moreover, the biological similarities of MBL with CLL-like and CLL phenotypes are demonstrable through other levels of scientific evidence: first, the protein expression profile is almost identical between them and, second, some chromosomal abnormalities frequently found in CLL patients are also present in individuals with CLL-like MBL.^16^ However, despite this biological relationship, a recent paper by Rossi et al.^17^ showed that MBL and CLL are clinically different conditions, given that individuals with MBL have a more favorable clinical course than do patients with the diagnosis of Rai 0 CLL.

With regard to clinical progression, Rawstron et al.^18^ followed up 185 subjects with clinical MBL for more than six years (median follow-up period of 6.7 years) and showed that CLL requiring treatment developed at a rate of 1.1% per year, mainly in subjects with clinical MBL, which is similar to the rate of progression to myeloma seen in patients with monoclonal gammopathy of undetermined significance. On the other hand, although limited data are available, progression among individuals with population-screening MBL is rarely seen in the practice of authors at different centers with wide experience of the management of MBL.^18^ Moreover, new studies are providing compelling evidence that not only the clinical characteristics but also the biology of population-screening MBL differ from that of clinical MBL. Thus, Dagklis et al.^8^ have shown that the usage of immunoglobulin heavy chain variable region (IGHV) is different between clinical and population-screening MBL, which means that the two conditions are probably biologically distinct entities.

**Figure 1:**
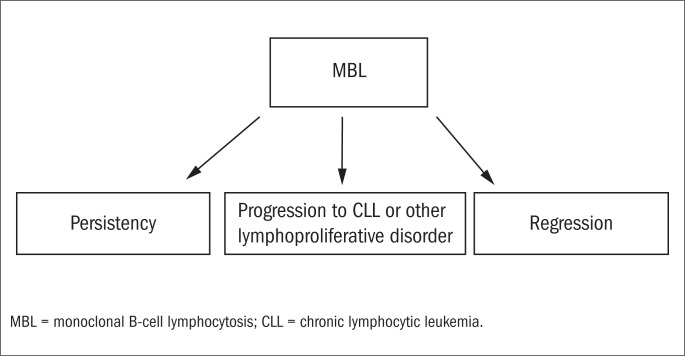
Possible outcomes from monoclonal B-cell lymphocytosis (MBL).

Hence, it is possible to infer that individuals with population-screening MBL are unlikely to be at any substantially higher risk of developing CLL than observed among the general population.^18^ This means that, in a general manner, MBL is not necessarily a pre-malignant condition. This conception is reinforced by studies showing that although MBL was originally thought to be exclusively monoclonal, both oligoclonal^19^ and polyclonal^8,14^ cases have been described. Thus, MBL can now be considered to be an entity with three possible different outcomes (Figure 1).

In this context, the distinction between a malignant and a premalignant condition is best made based on the individual's risk of an adverse clinical outcome.^20^ Currently, only B-cell counts in peripheral blood can be used to make a distinction among patients with monoclonal B-cells with regard to the necessity of starting some kind of therapy in the light of a risk of death. Thus, at the present time, only limited data are available for ascertaining whether prognostic factors classically associated with CLL outcomes, as determined by flow cytometry (CD38, CD49d and ZAP-70), cytogenetics (del 17 and del 11q) or molecular biology methods (*IGHV* rearrangement), can be used to predict the risk of MBL progression.

## DIAGNOSIS EVALUATION AND CLINICAL MANAGEMENT

The differential diagnosis between MBL, CLL and another related condition known as small lymphocytic lymphoma is based on peripheral blood B-cell counts and physical examinations on patients. MBL is characterized by an absolute peripheral blood B-cell count lower than 5 x 10^9^ cells/l and absence of lymphadenopathy and hepatosplenomegaly. Small lymphocytic lymphoma is also characterized by an absolute peripheral blood B-cell count lower than 5 x 10^9^ cells/l, but the presence of lymph nodes, liver or spleen enlargement is typical of this disease. CLL is defined by the presence of more than 5 x 10^9^ B-cells/l in peripheral blood. Lymphadenopathy and hepatosplenomegaly are found to varying degrees in patients with CLL (Table 1).^15^ Patients with a diagnosis of CLL, small lymphocytic lymphoma, clinical CLL-like MBL or atypical/non-CLL-like MBL should undergo a complete evaluation by a hematologist.

**Table 1:** Differential diagnosis: monoclonal B-cell lymphocytosis (MBL), small lymphocytic lymphoma (SLL) and chronic lymphocytic leukemia (CLL) (adapted from Shanafelt et al.)^15^

	Peripheral blood B-cells count < 5 × 10^9^/l	Lymphadenopathy or hepatosplenomegaly
MBL	Yes	No
SLL	Yes	Yes
CLL	No	Yes or No

The clinical management for individuals with MBL differs according to whether the patient presents population-screening MBL, clinical CLL-like MBL or atypical/non-CLL-like MBL. In practical terms, general clinicians should follow up individuals with population-screening MBL, given that these individuals have completely normal blood count and, in particular, that clinical experience has shown that progression in this group is very rare. For such individuals, close monitoring is not necessary and an annual examination with a complete blood count is sufficient and appropriate.^15^

As previously stated, patients with clinical CLL-like MBL have a risk of progression to CLL requiring treatment that is 1.1% per year.^18^ Based on this low risk, an annual follow-up with a complete blood count made by a hematologist is recommended. These patients should be counseled to pay attention to specific symptoms such as lymph node enlargement, nighttime sweating, extreme fatigue and weight loss, given that these clinical findings could be the first evidence of disease progression.

Patients with atypical or non-CLL-like MBL require a more thorough evaluation. In cases of lymph node enlargement found by computed tomography scans, these patients are best classified as having a non-Hodgkin lymphoma subtype, which should be subclassified based on complementary examinations (immunohistochemistry, cytogenetics etc). For such individuals, close clinical and laboratory monitoring is mandatory. Table 2 summarizes the recommendations for evaluation and follow-up of MBL in routine practice.

**Table 2:** Recommendations for evaluation and follow-up of monoclonal B-cell lymphocytosis(MBL). Adapted from Shanafelt et al.^15^

Recommendations	Population-screening MBL	Clinical CLL-like MBL	Atypical/non-CLL-like MBL
**(1) Diagnostic evaluation**			
History* and physical examination	Yes	Yes	Yes
CBC and immunophenotyping	Yes	Yes	Yes
FISH with t(11;14) probe	No	No	Yes†
CT scans on chest/abdomen/pelvis	No	No	Yes
Bone marrow biopsy	No	No	Yes
CLL prognostic testing	No	No	No
**(2) Follow-up**‡			
Risk of progression requiring therapy	Low	1-2%/year	Undefined
History and physical examination	Routine medical care	Annual	3-12 months
CBC	Annual	6-12 months	6-12 months
CT scans on chest/abdomen/pelvis	No	No	Clinical judgment

CLL, chronic lymphocytic leukemia; CBC, complete blood count; FISH, fluorescence *in situ* hybridization; CT, computed tomography.

* Fever, nighttime sweating, weight loss and fatigue

† For patients with CD5+/CD23− MBL

‡ For the rare individuals fulfilling the criteria for MBL who have an immunophenotype and cytogenetic evaluation suggestive of mantle-cell lymphoma, i.e. a “CD5+/CD23− MBL” case with the presence of t(11;14), or another aggressive non-Hodgkin lymphoma, the clinical follow-up should be done every 3-6 months with CT imaging at least every 6 months. For patients with MBL of atypical-CLL phenotype or non-CLL phenotype whose immunophenotype is consistent with a more indolent non-Hodgkin lymphoma subtype, follow-up every 6-12 months is recommended and the frequency of follow-up imaging requires clinical judgment.

## CONCLUSIONS

Monoclonal B-cell lymphocytosis is a very common clinical condition that is frequently found in asymptomatic subjects. It is biologically related to chronic lymphocytic leukemia and, in some cases, can be found prior to the diagnosis of CLL, although, at the present time, MBL cannot necessarily be considered to be a pre-leukemic condition.

The prevalence of MBL is at least 100 times higher than that of CLL. Thus, given that both availability of automated blood cell counters and access to flow cytometry tests are more widespread nowadays, it is not surprising that clinicians may unexpectedly find MBL cases during their daily practice. Within this scenario, it is important to recognize situations in which patients with a diagnosis of MBL can be followed up in general clinicians’ offices, versus situations in which they must be referred to a hematologist.

For diagnostic purposes, monoclonal B-cell lymphocytosis should be distinguished from chronic lymphocytic leukemia and small lymphocytic lymphoma. Moreover, it is essential to classify individual cases as “population-screening MBL” or “clinical MBL” and, furthermore, based on immunophenotyping, as “MBL with CLL-like phenotype”, “MBL with atypical CLL phenotype” or “MBL with non-CLL phenotype”, given that the clinical management for these patients is strongly dependent on this classification.

## LITERATURE SEARCH

We conducted a search in the literature regarding MBL in the five databases, using the term monoclonal B-cell lymphocytosis, and limiting the search to papers published in English over the last 20 years, including all types of articles (clinical trials, editorials, letters, meta-analyses, practical guidelines or randomized controlled trials). Articles with relevant information about the epidemiological, biological and clinical aspects of MBL have been included in the reference list of this paper. The results from the search are shown in Table 3.

**Table 3:** Database search results

Database	Search limits	Results
PubMed	Term: monoclonal B-cell lymphocytosis	19 reviews
	Types of articles: all.	3 letters
	Language: English	2 prospective cohort studies
	Period: 1990-2010	1 editorial
SciElo	Term: monoclonal B-cell lymphocytosis	1 review
Cochrane Library	Term: monoclonal B-cell lymphocytosis	None
Embase	Term: monoclonal B-cell lymphocytosis	None
Lilacs	Term: monoclonal B-cell lymphocytosis	None
